# Community Structure, Abundance and Potential Functions of Bacteria and Archaea in the Sansha Yongle Blue Hole, Xisha, South China Sea

**DOI:** 10.3389/fmicb.2019.02404

**Published:** 2019-10-18

**Authors:** Hui He, Lulu Fu, Qian Liu, Liang Fu, Naishuang Bi, Zuosheng Yang, Yu Zhen

**Affiliations:** ^1^College of Marine Life Sciences, Ocean University of China, Qingdao, China; ^2^Laboratory for Marine Ecology and Environmental Science, National Laboratory for Marine Science and Technology, Qingdao, China; ^3^Center of Deep Sea Research, Institute of Oceanology, Chinese Academy of Sciences, Qingdao, China; ^4^Key Laboratory of Experimental Marine Biology, Institute of Oceanology, Center for Ocean Mega-Science, Chinese Academy of Sciences, Qingdao, China; ^5^Key Laboratory of Marine Environment and Ecology, Ministry of Education, Qingdao, China; ^6^Sansha Trackline Institute of Coral Reef Environment Protection, Sansha, China; ^7^College of Marine Geosciences, Ocean University of China, Qingdao, China; ^8^College of Environmental Science and Engineering, Ocean University of China, Qingdao, China

**Keywords:** microbial ecology, the Sansha Yongle Blue Hole, extremophiles, 16S rRNA gene sequencing, community characteristics

## Abstract

The Sansha Yongle Blue Hole is the deepest blue hole in the world and exhibits unique environmental characteristics. In this paper, Illumina sequencing and qPCR analysis were conducted to obtain the microbial information in this special ecosystem. The results showed that the richness and diversity of bacterial communities in the hole was greater than those of archaeal communities, and bacterial and archaeal communities were dominated by *Proteobacteria* and *Euryarchaeota*, respectively. Temperature and nitrate concentration significantly contributed to the heterogeneous distribution of major bacterial clades; salinity explained most variations of the archaeal communities, but not significant. A sudden increase of bacterial 16S rRNA, archaeal 16S rRNA, ANAMMOX 16S rRNA, *nirS* and *dsrB* gene was noticed from 90 to 100 m in the hole probably due to more phytoplankton at this depth. Sulfur oxidation and nitrate reduction were the most abundant predicted ecological functions in the hole, while lots of archaea were predicted to be involved in aerobic ammonia oxidation and methanogenesis. The co-occurrence network analysis illustrated that a synergistic effect between sulfate reduction and sulfur oxidation, and between nitrogen fixation and denitrification, a certain degree of coupling between sulfur and nitrogen cycle was also observed in the hole. The comparisons of bacterial and archaeal communities between the hole and other caves in the world (or other areas of the South China Sea) suggest that similar conditions are hypothesized to give rise to similar microbial communities, and environmental conditions may contribute significantly to the bacterial and archaeal communities.

## Introduction

Anchialine caves are unique geomorphological units found on karst, volcanic islands and peninsular coastlines around the world that are mostly isolated from each other and have high scientific research and social value. Many anchialine caves were formed during the Quaternary period (approximately 2.5 million years ago to present) because of cyclical sea-level changes (Mylroie and Mylroie, 2011; Pérez-Moreno et al., 2016). Therefore, these anchialine caves can be hundreds to thousands of years old and contain detailed records of environmental change and landscape evolution (Sullivan et al., 2016). To date, many anchialine caves have been partially explored, including the Saipan Blue Hole (the Pacific Ocean), the Dahab Blue Hole (Egypt), the Faanu Madugau’s Blue Hole (the Indian Ocean), the Gozo Blue Hole (the Mediterranean Sea) and the Dean’s Blue Hole (the Bahama Islands). The water exchange of anchialine caves with other marine habitats is severely restricted, resulting in the relatively independent environments and special physical-chemical parameters in these caves (Iliffe and Kornicker, 2009; Pakes, 2013; Pérez-Moreno et al., 2016). Little or no photosynthetic oxygen production, stratified water columns and restricted vertical mixing contribute to the anoxic or micro-oxic environment and hydrogen sulfide in anchialine caves (Iliffe, 2000; Seymour et al., 2007; Becking et al., 2011; Gonzalez et al., 2011).

The unique abiotic factors such as dissolved oxygen (DO) concentration, water stratification and temperature in anchialine caves make them natural laboratories for studying marine biodiversity and biological adaptation and evolution. A number of studies on the phytoplankton, zooplankton and benthos in anchialine caves have been performed (Iliffe, 2000, 2004; Bishop et al., 2004; Gerovasileiou et al., 2016), resulting in the description of numerous new species and cognitions previously unknown to science (Iliffe, 2002; Pérez-Moreno et al., 2016). Yager (1981) isolated four new individuals, namely, *Remipedia* (new class), *Speleonectidae* (new family), *Speleonectes* (new genus) and *Speleonectes lucayensis* (new species) from Lucayan Cavern on Grand Bahama Island, Bahamas. The new genus *Nanocopia*, which was considered to be more closely related to *Platycopia*, was isolated from a marine cave on Bermuda by Fosshagen and Iliffe (1988). Furthermore, the study of organisms in anchialine caves has broadened our understanding; for example, the mouthparts of adult male *Centropages orsinii* Giesbrecht, 1889 were first described and illustrated in a study of an anchialine cave in Vanuatu (Boxshall and Jaume, 2012). Due to the absence of light and poor supplies of easily degradable organic matter, anchialine caves are considered to be extreme environments for life (Northup and Lavoie, 2001; Krstulović et al., 2013). The microbial diversity, abundance and activity in such environments become a popular topic of research. Extensive studies on the microbial diversity and abundance in anchialine caves began to emerge in recent years (Seymour et al., 2007; Garman et al., 2011; Krstulović et al., 2013; Davis and Garey, 2018). Based on the 16S rRNA gene, many species in the Jewfish Sink were found to be similar to those in other anoxic environments, and the microbial community in the sink was rich in species likely to be involved in the sulfur, nitrogen and methane cycles (Garman et al., 2011). Krstulović et al. (2013) studied the bacterial abundance and diversity in two anchialine caves (Bjejajka Cave and Lenga Pit) located on Mljet Island and found that the microbes in both caves exhibited lower diversity, higher abundances and unique compositions, with *Epsilonproteobacteria* representing the most abundant group. In the Hospital Hole, there are unique microbial communities in each layer, and sulfur oxidation and nitrogen reduction, which are often coupled, were the predominant ecological functions (Davis and Garey, 2018).

The Sansha Yongle Blue Hole (111°46′06′′E, 16°31′30′′N) in the Xisha Islands is currently the deepest blue hole (∼300 m) in the world. Unlike other marine blue holes that have been found throughout the world, the Sansha Yongle Blue Hole is located in the continental slope of the deeper water in the South China Sea (SCS), and there are many shallow reefs and submerged reefs around it (Gai, 2016; Liu et al., 2017). Therefore, the Yongle Blue Hole has high scientific research value; however, the investigation of this hole is only in the initial stage. The Yongle Blue Hole belongs to an anchialine cave system, which shows noticeable marine as well as terrestrial influences (Stock et al., 1986; Yao et al., 2018). DO in the hole decreases sharply and reaches 0 mg ⋅ L^–1^ at approximately 90 m, and the hole has no large-scale connection with adjacent oceans (Bi et al., 2018). In March 2017, the zooplankton communities in water from the Yongle Blue Hole and the outer reef slope were studied by Chen’s group (Chen et al., 2018). For planktonic larvae in water from the hole, 41 species and 14 groups were identified, while 124 species and 20 groups were found in water from the outer reef slope; the zooplankton showed a diel vertical distributional difference, and *Oithona attenuata* dominated during both the daytime and nighttime, followed by *O. rigida* and *Scolecithricella longispinosa*. Anchialine caves are characterized by lack of light or completely darkness, relatively constant air and water temperature, and poor supplies of easily degradable organic matter (Krstulović et al., 2013). Studies have shown that the microbial communities within caves are similar to others, verifying the hypothesis that the microbial assemblages are selected by the environments. Although 14 km apart, similar microbes, such as *Arcobacter*, *Sulfurimonas*, *Desulfobacterium*, *Desulfofaba*, and *Desulfosarcina*, were present both in Jewfish Sink and Hospital Hole (Davis and Garey, 2018). Marine blue holes differ from other marine habitats because of high sulfide concentrations, low oxygen concentrations and restricted vertical mixing (Gonzalez et al., 2011). Researchers have found that the compositions of zooplankton and archaea within the Yongle Blue Hole differ greatly from other areas of the SCS, indicating different communities (Chen et al., 2018; Zhen et al., 2018). In the present study, the composition and distribution of bacterial and archaeal communities in the Yongle Blue Hole are studied to gain a comprehensive understanding of the microbial communities within this special habitat, which will fill the gap in microbial studies of the Yongle Blue Hole. Moreover, comparisons of the bacterial and archaeal communities in the Yongle Blue Hole and other anchialine caves, as well as comparisons of the bacterial and archaeal communities in the Yongle Blue Hole and other areas of the SCS are also discussed in this paper.

## Materials and Methods

### Sampling

Environmental samples were collected from the Yongle Blue Hole and the outer reef slope from 14 March to 22 March, 2017. A total of 14 seawater samples were collected, including 10 samples (YL0m, YL10m, YL20m, YL40m, YL60m, YL80m, YL90m, YL100m, YL150m, YL180m) from the Yongle Blue Hole and 4 samples (RS0m, RS50m, RS150m, RS200m) from the outer reef slope. The seawater samples (5 L) were prefiltered through a 75-μm mesh net to remove large organisms and particles and then filtered through 0.22-μm polycarbonate membranes (Millipore Corporation, United States). The membranes were stored in liquid nitrogen until DNA extraction. The environmental factors at the sampling stations such as chlorophyll *a* content (Chla) and DO were recorded with a Conductivity, Temperature and Depth (CTD) profiler (SBE 19 Plus, Sea-Bird Electronics, Inc.) (Table 1).

**TABLE 1:** The environmental factors at the 14 sampling stations.

**Sample**	**Depth (m)**	**Temperature (°C)**	**Salinity (PSU)**	**pH**	**Turbidity (FTU)**	**Chla (μg ⋅ L^–1^)**	**DO (mg ⋅ L^–1^)**
YL0m	0	27.47	33.40	8.14	0.18	0.13	6.34
YL10m	10	25.95	33.40	7.99	0.38	0.82	4.62
YL20m	20	25.23	33.40	7.96	0.27	1.63	4.34
YL40m	40	24.64	33.50	8.01	0.16	0.30	5.09
YL60m	60	24.28	33.50	8.04	0.13	0.10	5.11
YL80m	80	23.43	33.60	7.99	0.54	0.04	4.28
YL90m	90	21.11	34.30	7.66	0.58	0.07	0.83
YL100m	100	18.99	34.40	7.55	0.85	0.11	0.05
YL150m	150	15.75	34.50	7.43	0.57	0.24	0.00
YL180m	180	15.68	34.50	7.26	0.17	0.00	0.00
RS0m	0	–	33.50	8.02	–	–	–
RS50m	50	–	34.00	7.99	–	–	–
RS150m	150	–	34.80	7.81	–	–	–
RS200m	200	–	34.70	7.75	–	–	–

*YL^∗∗^ and RS^∗∗^ represent the samples from the Sansha Yongle Blue Hole and the outer reef slope, respectively. –, not available.*

### Nucleic Acid Isolation and Illumina Sequencing Analysis

The membranes were cut into small pieces and put into sterile 2.0-mL tubes. Then, 1000 μL of cetyltrimethyl ammonium bromide (CTAB) was added into each tube. After incubation at 65°C for 2 h, each tube was vortexed and centrifuged. Then, 950 μL of supernatant was transferred into new tubes, extracted with equal amounts of phenol-chloroform-isoamyl alcohol (25:24:1), and centrifuged at 10000 *g* for 10 min. The supernatant was transferred into new tubes, extracted with equal amounts of chloroform-isoamyl alcohol (24:1), and then centrifuged at 10000 *g* for 10 min. After being transferred into new 1.5-mL tubes, the supernatant was mixed with three-quarters of supernatant of isoamyl alcohol for precipitation and then centrifuged at 10000 *g* for 10 min. The final aqueous layer was recovered, washed and precipitated twice with 1 mL of 75% alcohol, and resuspended in 51 μL of sterile double-distilled water.

The bacterial and archaeal community characteristics were studied by using Illumina HiSeq 2500 sequencing of the 16S rRNA gene. The fragments were amplified using the specific primer pairs 341F/806R and U519F/806R for bacteria and archaea, respectively (Table 2). The 30-μL reaction system contained 15 μL of Phusion High-Fidelity PCR Master Mix (2×) (New England Biolabs), 0.2 μM primers and approximately 10 ng of template DNA. The reactions were held at 98°C for 1 min to denature the DNA, followed by 30 cycles of 98°C for 10 s, 50°C for 30 s and 72°C for 30 s and a final step at 72°C for 5 min. After amplification, the amplicons were analyzed via 2% agarose gel electrophoresis, then mixed in equal-density ratios and purified with a GeneJET Gel Extraction Kit (Thermo Scientific). Sequencing libraries were generated using an Illumina TruSeq DNA PCR-Free Library Preparation Kit (Illumina, United States), and index codes were added. The libraries were sequenced on an Illumina HiSeq 2500 platform by the Novogene Bioinformatics Technology Company (Beijing, China).

**TABLE 2:** Primers used in this study.

**Target gene**	**Primer**	**Sequence (5′-3′)**	**References**
Bacterial 16S rRNA gene	341F	CCT AYG GGR BGC ASC AG	Michelsen et al., 2014
	806R	GGA CTA CNN GGG TAT CTA AT	
Archaeal 16S rRNA gene	U519F	CAG YMG CCR CGG KAA HAC C	Porat et al., 2010
	806R	GGA CTA CNS GGG TMT CTA AT	
ANAMMOX 16S rRNA gene	Amx368F	TTC GCA ATG CCC GAA AGG	Schmid et al., 2005
	Amx820R	AAA ACC CCT CTA CTT AGT GCC C	
*nirS*	cd3aF	GTS AAC GTS AAG GAR ACS GG	Throbäck et al., 2004
	R3cd	GAS TTC GGR TGS GTC TTG A	
*dsrB*	DSRp2060F	CAA CAT CGT YCA YAC CCA GGG	Geets et al., 2006
	DSR4R	GTG TAG CAG TTA CCG CA	

### Bioinformatic Analysis

The paired-end reads generated from the Illumina HiSeq 2500 platform were processed by trimming the barcodes and primers and then merged using Fast Length Adjustment of SHort reads (FLASH). Reads shorter than 200 base pairs, with an average quality score lower than 20 and with any ambiguous bases, were removed. UPARSE (Edgar, 2013) was used to cluster the clean data into operational taxonomic units (OTUs), with a 97% similarity cutoff. The most common sequences in each OTU were selected as representative sequences. Taxonomic assignments were annotated based on an 80% confidence level with the Greengenes and SILVA databases for bacteria and archaea, respectively. Reads which did not match any sequences in the database were clustered into the unclassified group. Community richness (Chao1 estimator), diversity (Shannon index) and Good’s coverage were calculated with Quantitative Insights into Microbial Ecology (QIIME) (version 1.9.0). Annotation of Prokaryotic Taxa (FAPROTAX) was used to predict the ecological functions of bacterial and archaeal communities (Louca et al., 2016). Microbial co-occurrence network analysis was conducted using R package psych. To reduce complexity, the bacterial genus with a relative abundance of more than 0.1% and all archaeal genus were selected to generate the co-occurrence patterns. A Spearman’s coefficient of greater than 0.6 and a significance level of less than 0.05 indicated a significant correlation. Finally, a network diagram was generated by Gephi software (version 0.9.2) (Bastian et al., 2009). Redundancy analysis (RDA) was performed to explore the correlations between environmental factors and microbial communities with CANOCO for Windows (version 4.5) (ter Braak and Šmilauer, 2002). Pearson’s correlation analysis was employed between environmental factors and microbial abundance by SPSS statistical software (version 19.0). The raw data generated by Illumina sequencing were deposited into the Sequence Read Archive (SRA) database under the accession numbers SRP152110 (bacterial 16S rRNA gene) and SRP152191 (archaeal 16S rRNA gene).

### Quantitative PCR

The bacterial 16S rRNA gene, archaeal 16S rRNA gene, anaerobic ammonia-oxidizing bacteria (ANAMMOX) 16S rRNA gene, denitrifying bacteria cd1-nitrite reductase gene (*nirS*) and sulfate-reducing bacteria (SRB) dissimilatory sulfite reductase β subunit gene (*dsrB*) were amplified with 341F/806R, U519F/806R, Amx368F/Amx820R, cd3Af/R3cd and DSRp2060F/DSR4R, respectively (Table 2). All qPCR assays were performed in triplicate with an ABI PRISM^®^ 7500 Sequence Detection System (Applied Biosystems, United States) using SYBR Green I. Each 20-μL qPCR contained 10 μL of FastStart Universal SYBR Green Master Mix (ROX) (Roche, Germany), 0.3 μM primers, 0.2 μg ⋅ μL^–1^ bovine serum albumin (BSA) and 2.0 μL of DNA. The qPCR amplification conditions of bacterial, archaeal and ANAMMOX 16S rRNA gene were as follows: 95°C for 10 min followed by 40 cycles of 30 s at 95°C and 1 min at 58°C. As for another two genes, the procedure was employed as follows: an initial denaturation at 95°C for 10 min, followed by 40 cycles of 30 s at 95°C, 30 s at 53°C for *nirS* gene (or 40 s at 55°C for *dsrB* gene) and 45 s at 72°C. To obtain a melting curve, a melting stage was performed after the amplification cycles. Standard curves were generated with the target standard plasmids. The abundance of the five target genes listed above was examined using the mentioned above qPCR protocols. In addition to the template DNA, each reaction included serially diluted plasmids and negative controls to ensure the qPCR assay was uncontaminated and stable. The data were analyzed with the ABI PRISM^®^ 7500 software (Applied Biosystems, version 1.3.1).

## Results

### Bacterial 16S rRNA Gene Analysis

In this study, a total of 594717 high-quality bacterial 16S rRNA gene sequences, ranging from 27420 to 53731 at each station, were obtained from the 14 seawater samples, with an average sequence length of 414 base pairs (Table 3). A total of 2538 OTUs were classified based on a 97% sequence similarity, and each station possessed 580–1052 OTUs. Good’s coverage ranged from 98.75 to 99.85%, indicating that the bacterial 16S rRNA gene sequences retrieved from these 14 seawater samples represented the majority of the bacterial communities in the studied areas. The highest bacterial diversity and the lowest bacterial richness were both found in YL60m. The highest bacterial richness was found in YL0m, while the lowest bacterial diversity was observed in YL150m. Neither the Chao1 estimator nor the Shannon index showed remarkable differences between the hole and the outer reef slope (*P* > 0.05).

**TABLE 3:** The richness estimator, diversity index and Good’s coverage of bacterial and archaeal communities.

**Sample**	**Bacteria**					**Archaea**				
	**Reads**	**OTUs**	**Chao1**	**Shannon**	**Good’s coverage (%)**	**Reads**	**OTUs**	**Chao1**	**Shannon**	**Good’s coverage (%)**
YL0m	46840	1052	1265.62	5.15	98.75	27288	433	896.30	4.44	98.70
YL10m	46761	1016	1211.31	5.50	98.80	28637	377	744.23	3.60	98.92
YL20m	44616	859	1122.30	4.97	98.92	37461	155	366.12	1.66	99.41
YL40m	41953	772	998.54	3.88	99.04	44976	144	360.19	0.72	99.46
YL60m	46993	649	661.27	7.94	99.85	33599	133	342.15	0.84	99.43
YL80m	27995	798	1105.12	5.86	98.98	–	–	–	–	–
YL90m	27420	630	979.21	4.68	99.10	–	–	–	–	–
YL100m	46961	740	921.74	4.62	99.06	28960	110	278.10	0.58	99.55
YL150m	47448	580	764.60	3.35	99.18	36243	139	390.28	1.11	99.37
YL180m	39295	916	1129.98	5.90	98.97	31019	134	388.83	1.71	99.40
RS0m	53731	646	763.48	3.52	99.25	30878	134	426.30	2.42	99.35
RS50m	44238	703	912.29	4.69	99.14	23502	200	464.30	4.51	99.32
RS150m	43096	616	744.61	5.11	99.30	26947	200	463.17	4.78	99.31
RS200m	37370	693	889.39	6.14	99.15	17147	218	572.97	4.96	99.14

*–, not available.*

**FIGURE 1:**
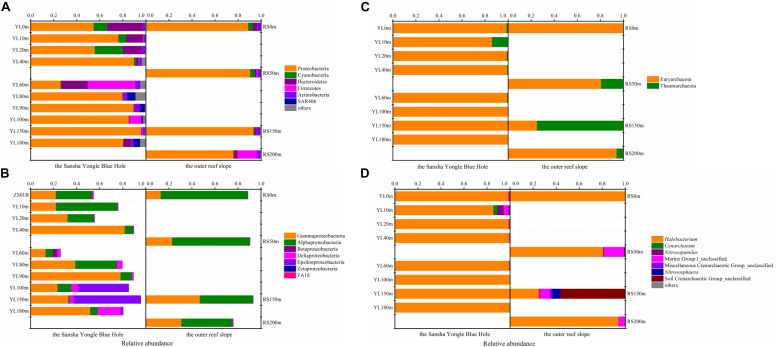
Relative abundance of the dominant bacteria and archaea at different levels in the water from the Sansha Yongle Blue Hole and the outer reef slope. **(A)** Relative abundance of the dominant bacteria at the phylum level. **(B)** Relative abundance of the *Proteobacteria* at the class level. **(C)** Relative abundance of the dominant archaea at the phylum level. **(D)** Relative abundance of the dominant archaea at the genus level.

In total, 37 different bacterial phyla were found across all samples. The dominant phylotypes were those with a frequency of >1% within a sample (Galand et al., 2009). In this study, the dominant phyla were *Proteobacteria* (26.03–95.74%), *Cyanobacteria* (0.04–24.37%), *Bacteroidetes* (0.25–30.52%), *Firmicutes* (0.23–41.06%), *Actinobacteria* (1.01–4.61%), and *SAR406* (0.01–7.04%), which accounted for 94.92–99.82% of the total sequences (Figure 1A). *Proteobacteria* was the dominant phylum across all samples, with the exception of YL60m, where *Firmicutes* was the most abundant phylum. Within *Proteobacteria*, *Gammaproteobacteria* and *Alphaproteobacteria* dominated across all samples except YL100m and YL150m, where *Epsilonproteobacteria* was the most abundant class (Figure 1B). *Gammproteobacteria* was more abundant in the hole, while *Alphaproteobacteria* was dominant in the water from the outer reef slope and displayed a decreasing trend with depth. Significant differences in the abundances of *Alphaproteobacteria* and *Epsilonproteobacteria* were observed between the hole and the outer reef slope (*P* < 0.05).

At the order level, *Gammaproteobacteria* was dominated by the orders *Vibrionales*, *Alteromonadales*, *Thiotrichales*, *Oceanospirillales*, *Pseudomonadales* and *Thiohalorhabdales*. The mean abundance of *Vibrionales*, *Oceanospirillales*, *Thiotrichales* and *Thiohalorhabdales* in the water from the hole was greater than that from the outer reef slope, whereas the mean abundance of *Alteromonadales* and *Pseudomonadales* was greater in the water from the outer reef slope (data not shown). Among these dominant orders within *Gammaproteobacteria*, only the abundance of *Alteromonadales* showed a remarkable difference between the hole and the outer reef slope (*P* < 0.05). For *Alphaproteobacteria*, the dominant orders were *Rhodobacterales*, *Sphingomonadales*, *Rickettsiales*, *Rhizobiales*, and *Rhodospirillales*, and the abundance of the last two orders increased with depth in the outer reef slope. Significant differences in the abundance of *Sphingomonadales* and *Rhodobacterales* were observed between the hole and the outer reef slope (*P* < 0.05). Heterogeneous distributions of other dominant orders along the depth profiles were also observed. For example, *Desulfarculales* displayed an increasing trend with depth in the hole, while *Synechococcales* exhibited the opposite trend; *Bacillales* showed an increasing trend with depth in the outer reef slope, whereas *Synechococcales* was observed to have a decreasing trend. At the family level, *Desulfarculaceae*, *Desulfobacteraceae* and *Desulfobulbaceae* increased with depth in the hole, and their abundance in the hole was much greater than that in the outer reef slope. In our study, the abundance of the above mentioned three bacteria in the portion of the water column below 90 m was significantly greater than that in the portion of the water columns above 90 m (*P* < 0.05), most likely because it became anaerobic conditions and sulfate reduction process might be more frequent in these water columns (Bi et al., 2018; Sun et al., 2018).

**FIGURE 2:**
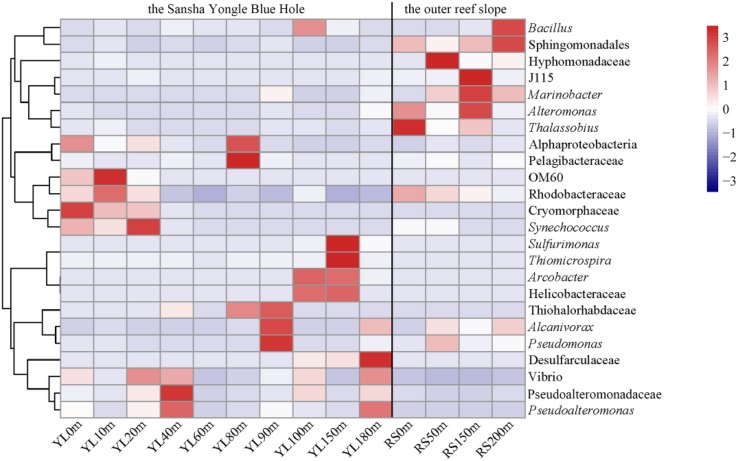
Heatmap of the dominant bacteria at the genus level in the water from the Sansha Yongle Blue Hole and the outer reef slope. Clades are given at a higher taxonomic level if the sequences could not be assigned to a genus.

For the 24 dominant genera, a detailed abundance pattern across all samples at the genus level was illustrated by a heatmap (Figure 2). The dominant bacterial genera in the water from the hole and the outer reef slope were quite different. An unknown genus within *Sphingomonadales* was the dominant genus in the water from the outer reef slope, while its abundance was relatively low in the water from the hole, and a significant difference in its abundance was found between the hole and the outer reef slope (*P* < 0.05). For the bacterial communities in the hole, *Synechococcus* and *Cryomorphaceae_unclassified* were the dominant genera in the portions of the water column above 80 m; whereas, *Arcobacte*r, *Thiomicrospira* and *Sulfurimonas* occupied the dominant position in the portions of the water column below 80 m. In addition, an unknown genus with *Desulfarculaceae* was dominant in deep waters in the hole, especially in the water below 100 m.

### Archaeal 16S rRNA Gene Analysis

In total, 366657 high-quality sequences, ranging from 17147 to 44976 among the 12 seawater samples, were obtained for further analyses (Table 3), and their average length was 251 base pairs. In total, 683 OTUs were assigned based on a 97% sequence similarity, and 110–433 OTUs were identified in the 12 seawater samples. Good’s coverage was 99.28% on average, varying from 98.70 to 99.55%, indicating that the libraries represented the majority of the archaeal species in this natural habitat. The lowest archaeal richness and diversity were observed in YL100m, and the highest archaeal richness and highest diversity were found in YL0m and RS200m, respectively. For the richness and diversity of archaeal communities in the water from the outer reef slope, a tendency to increase with depth was observed. A significant difference in the Shannon index was found between the hole and the outer reef slope (*P* < 0.05), while the Chao1 estimator showed no remarkable difference (*P* > 0.05).

Two archaeal phyla, namely, *Euryarchaeota* and *Thaumarchaeota*, were found in this study. *Euryarchaeota* was the dominant phylum in most samples except RS150m, which was dominated by *Thaumarchaeota* (Figure 1C). An in-depth taxonomic analysis showed that *Halobacteria* in *Euryarchaeota* was prevalent except in RS150m, where *Soil Crenarchaeotic Group* (*SCG*) was the predominant class (data not shown). *Halobacterium*, which can be found in environments with a high salt concentration, was the most abundant genus in the present study except in RS150m, where *SCG_unclassified* was the dominant genus (Figure 1D) (Kennedy et al., 2001; Oren, 2008).

### Correlations Between Microbial Community Structure and Environmental Factors

**FIGURE 3:**
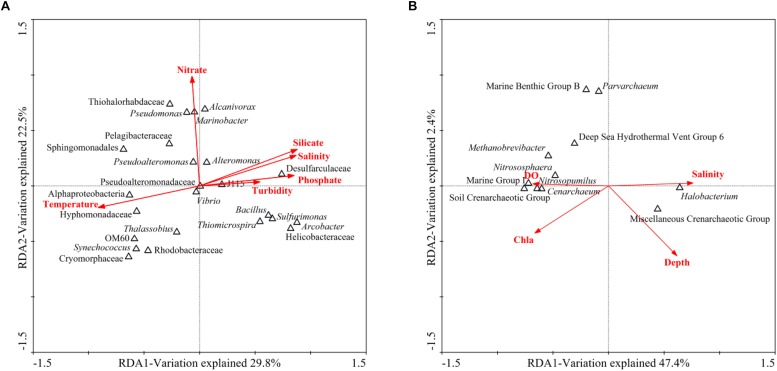
RDA ordination plots of the relationship between environmental factors and microbial community structure. **(A)** RDA targeting the dominant bacterial genera in the Sansha Yongle Blue Hole. **(B)** RDA targeting the archaeal genera in the Sansha Yongle Blue Hole.

BIO-ENV analysis was performed to identify the subset of environmental factors that could best explain the community variation across all samples. The nutrient (ammonium, nitrite, nitrate, silicate and phosphate) concentrations were provided by Yao’s group (Yao et al., 2018). Then, RDA was employed to reveal the correlations between microbial community structure and environmental factors. According to the BIO-ENV analysis, the bacterial communities were strongly correlated with temperature, turbidity, salinity, nitrate concentration, silicate concentration and phosphate concentration, while the archaeal communities were strongly related to depth, Chla, salinity and DO. For the bacterial communities in the hole, the first two RDA dimensions explained 52.3% of the total variance (Figure 3A). Monte Carlo permutations showed that temperature (*P* = 0.002) and nitrate concentration (*P* = 0.006) significantly contributed to the heterogeneous distribution of major bacterial clades. Temperature explained the majority of the variation, and *Alphaproteobacteria_unclassified* and *Hyphomonadaceae_unclassified* were more related to temperature. Nutrients (nitrogen, phosphorus, etc.) were also determining factors. For example, *Marinobacter* and *Pseudomonas* were positively related to nitrate concentration, while *Vibrio* was negatively related to nitrate concentration. For the archaeal communities in the hole, the first axis explained 47.4% of the total variance, while the second axis explained only 2.4% (Figure 3B). Although Monte Carlo permutations showed that none of the single environmental factors significantly contributed to the heterogeneous distribution of the archaeal clades (*P* > 0.05), salinity explained most of the variation. *Halobacterium* was positively related to salinity. DO was also an important determining factor. *Nitrososphaera*, *Nitrosopumilus* and *Cenarchaeum* as well as *Marine Group I_unclassified* (*MGI_unclassified*) and *SCG_unclassified* were positively correlated with DO.

### Predicted Ecological Functions Based on FAPROTAX

**FIGURE 4:**
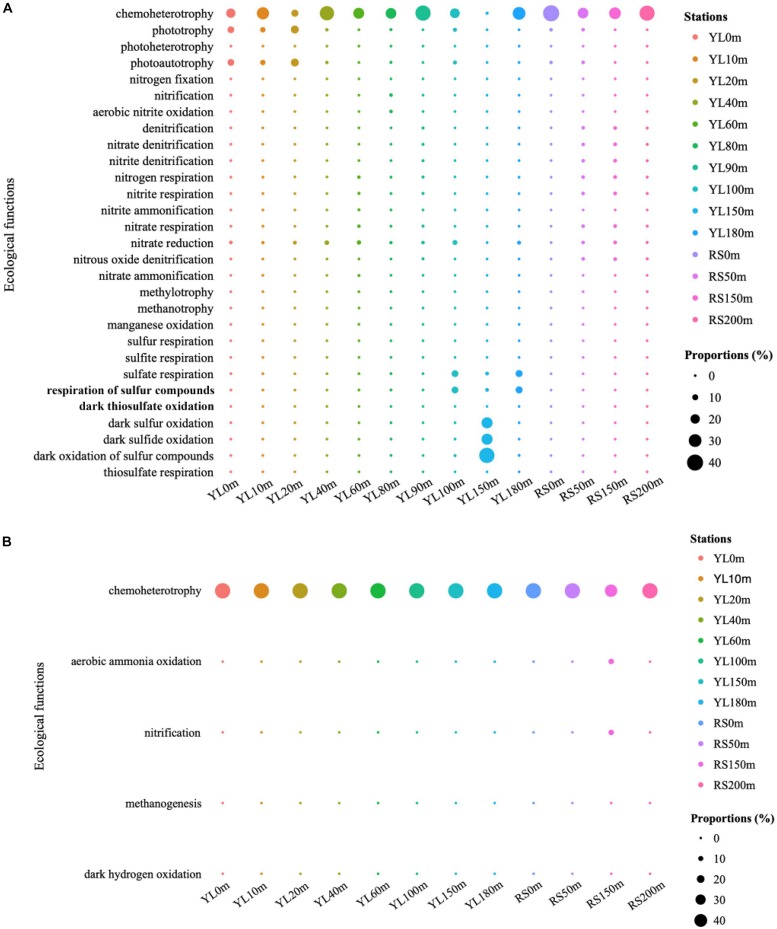
The predicted ecological functions of bacterial **(A)** and archaeal **(B)** communities based on 16S rRNA genes. Nitrogen fixation, nitrification, aerobic nitrite oxidation, denitrification, nitrate denitrification, nitrite denitrification, nitrogen respiration, nitrite respiration, nitrite ammonification, nitrate respiration, nitrate reduction, nitrous oxide denitrification, nitrate ammonification are parts of nitrogen cycle. Sulfur respiration, sulfite respiration, sulfate respiration, respiration of sulfur compounds, dark thiosulfate oxidation, dark sulfur oxidation, dark sulfide oxidation, dark oxidation of sulfur compounds and thiosulfate respiration are parts of sulfur cycle. Methylotrophy, methanotrophy and methanogenesis are parts of carbon cycle.

The predicted ecological functions of bacterial and archaeal communities were investigated by FAPROTAX. The bacterial community contained a high number of sequences assigned to chemoheterotrophy (Figure 4A). The representation of aerobic chemoheterotrophy in chemoheterotrophy was lowest in YL60m, where it was only 30.35%, but greater than 96.37% at other stations. Phototrophy and photoautotrophy were predicted to be greater in surface layers in the water from the hole; however, for phototrophy and photoautotrophy in bottom layers, a greater value in YL100m was also observed. In addition, many sequences were predicted to be involved in the sulfur cycle, such as sulfur oxidation and sulfate respiration. In this study, the abundance of sequences assigned to dark sulfur oxidation, dark sulfide oxidation and dark oxidation of sulfur compounds peaked in YL150m, and significant differences in the abundance of sequences assigned to the three ecological functions mentioned above were observed between the hole and the outer reef slope (*P* < 0.05). In addition, other processes in the sulfur cycle were represented; for example, the abundance of sequences assigned to sulfate respiration peaked in YL100m, YL150m and YL180m. In addition to the sulfur cycle, many sequences were predicted to play an important role in the nitrogen cycle. For example, a high proportion of bacteria involved in nitrate reduction were observed in this study. Sulfur oxidation and nitrate reduction were the most abundant ecological functions in the Sansha Yongle Blue Hole, consistent with the results obtained from the Hospital Hole in Florida (Davis and Garey, 2018).

For archaeal communities, the essential predicted function was chemoheterotrophy (Figure 4B). In addition, many sequences were predicted to have ecological functions involved in biogeochemical cycles, such as the nitrogen and carbon cycles. Ecological functions in nitrogen and carbon cycles, such as aerobic ammonia oxidation and methanogenesis, were also predicted to be present in the archaeal communities at relatively lower abundances.

### Microbial Co-occurrence Network Analyses

**FIGURE 5:**
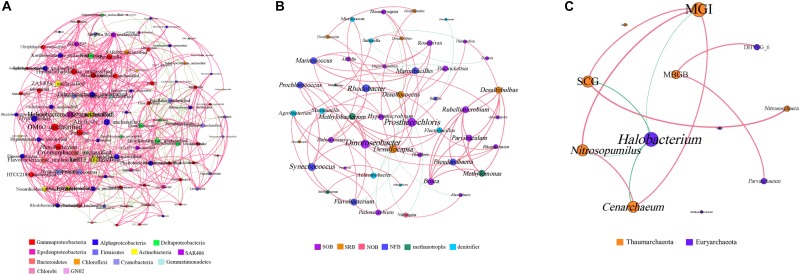
Co-occurrence network analyses of the bacterial genera **(A)**, the functional groups **(B)** and the archaeal genera **(C)** with a Spearman’s coefficient > 0.6 or <–0.6 and for which *P* < 0.05. The bacterial genera with relative abundance of more than 0.1% were selected. Each line represents a significant correlation between two genera. The red lines represent positive correlations, and the green lines represent negative correlations. The node size is proportional to the number of connections. SOB, sulfur-oxidizing bacteria; SRB, sulfate-reducing bacteria; NOB, nitrite-oxidizing bacteria; NFB, nitrogen-fixing bacteria. MGI, Marine Group I; MCG, Miscellaneous Crenarchaeotic Group; DHVEG_6, Deep Sea Hydrothermal Vent Group 6; SCG, Soil Crenarchaeotic Group; MBGB, Marine Benthic Group B; MGII, Marine Group II.

The bacterial genera with a relative abundance of more than 0.1% and all archaeal genera were selected to generate the microbial co-occurrence network in the hole. The bacterial network consisted of 100 nodes and 780 edges, and its average degree and average clustering coefficient were 15.12 and 0.57, respectively (Figure 5A). In the bacterial network, most correlations (77.25%) were positive. Members of *Firmicutes* formed a relatively independent cluster, showing a strong intra-phylum correlation. Members of *Alphaproteobacteria* and *Deltaproteobacteria* also showed a high proportion of intra-phylum positive correlations. Nevertheless, members of *Actinobacteria* and *Bacteroidetes* exhibited more positive correlations with other bacterial genus, especially with *Gammaproteobacteria*, *Alphaproteobacteria*, and *Deltaproteobacteria*. Furthermore, members of *Gammaproteobacteria* and *Alphaproteobacteria* were widely distributed in every cluster and exhibited more positive correlations with different bacterial genera, indicating that they may play the vital role in the hole. At the same time, we also analyzed the known bacterial genera to find their functional couplings in the hole (Figure 5B). A high degree of intrinsic association among SRB was observed in this study. Notably, we also found a significant positive correlation between SRB and sulfur-oxidizing bacteria (SOB). In addition, both SRB and SOB all showed significant correlation with nitrite-oxidizing bacteria (NOB). Moreover, there was also a significant positive correlation between nitrogen-fixing bacteria (NFB) and denitrifier in the hole. These results illustrated that sulfate reduction and sulfur oxidation process, nitrogen fixation and denitrification process may have a synergistic effect with each other, and a certain degree of coupling between sulfur and nitrogen cycle was also observed in the hole.

The archaeal co-occurrence network was much simpler than the bacterial one. A total of 11 pairs were found to be significantly correlated in 12 archaeal genera, among which 82.61% were significantly positive correlation (Figure 5C). The average degree of the archaeal network was 3.83, and the average clustering coefficient was 0.64. Specifically, both *MGI* and *Halobacterium* showed high incidence of correlations with other genera, suggesting that *MGI* and *Halobacterium* play a central role in the hole.

### Abundance of Bacterial 16S rRNA, Archaeal 16S rRNA and Functional Genes

**FIGURE 6:**
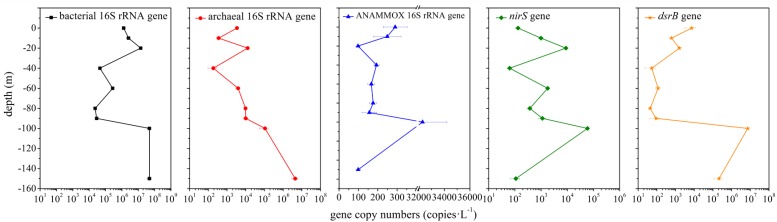
Quantitative analysis of bacterial 16S rRNA, archaeal 16S rRNA and functional genes (ANAMMOX 16S rRNA, *nirS* and *dsrB* genes) in the Sansha Yongle Blue Hole. The error bars represent the standard deviations of triplicate quantifications.

The abundance of bacterial 16S rRNA, archaeal 16S rRNA and functional genes (ANAMMOX 16S rRNA, *nirS* and *dsrB* gene) in the hole was examined via qPCR analysis. The abundance of bacterial 16S rRNA gene (2.32 × 10^4^ – 4.84 × 10^7^ copies ⋅ L^–1^) was always greater than that of archaeal 16S rRNA gene (1.85 × 10^2^ – 4.44 × 10^6^ copies ⋅ L^–1^) (Figure 6). The abundance of ANAMMOX 16S rRNA, *nirS* and *dsrB* gene ranged from 9.79 × 10^1^ to 3.23 × 10^4^ copies ⋅ L^–1^, 6.28 × 10^1^ to 5.92 × 10^4^ copies ⋅ L^–1^ and 4.38 × 10^1^ to 7.23 × 10^6^ copies ⋅ L^–1^, respectively. In the top 10 m, bacterial 16S rRNA and *nirS* gene copy numbers increased with depth, but archaeal 16S rRNA, ANAMMOX 16S rRNA and *dsrB* gene copy numbers decreased with depth. From a depth of 10 m to a depth of 20 m, bacterial 16S rRNA, archaeal 16S rRNA, *nirS* and *dsrB* gene copy numbers increased with depth, while ANAMMOX 16S rRNA gene copy numbers decreased with depth; however, from a depth of 20 m to a depth of 40 m, the copy numbers of these genes exhibited the opposite trend. A sudden and dramatic increase of the five target gene copy numbers was noticed from 90 to 100 m, probably owing to more phytoplankton at this depth in the hole (unpublished data). Then the functional gene copy numbers exhibited a tendency to decline significantly with depth. Environmental factors affected microbial abundance. For the abundance of bacterial 16S rRNA gene in the hole, temperature (*P* = 0.008, *r* = −0.809), turbidity (*P* = 0.05, *r* = 0.667), salinity (*P* = 0.022, *r* = 0.742), pH (*P* = 0.006, *r* = −0.824), DO (*P* = 0.016, *r* = −0.766), ammonium concentration (*P* = 0.026, *r* = 0.728), phosphate concentration (*P* = 0.013, *r* = 0.781) and silicate concentration (*P* = 0.003, *r* = 0.854) were the most significant factors. Depth (*P* = 0.039, *r* = 0.692), temperature (*P* = 0.022, *r* = −0.742), ammonium concentration (*P* = 0.000, *r* = 0.995), phosphate concentration (*P* = 0.000, *r* = 0.966) and silicate concentration (*P* = 0.006, *r* = 0.831) played a vital role in determining the abundance of archaeal 16S rRNA gene in the hole. Furthermore, turbidity was the determining factor for the abundance of ANAMMOX 16S rRNA (*P* = 0.045, *r* = 0.678) and *dsrB* (*P* = 0.040, *r* = 0.689) genes in the hole. No single environmental factor seemed to play a pivotal role in determining the abundance of *nirS* gene in the hole.

## Discussion

The Sansha Yongle Blue Hole is the deepest blue hole in the world, as well as the only known marine blue hole in China (Liu et al., 2017). This is the first study to present the diversity, composition and vertical variation of bacterial and archaeal communities in the Sansha Yongle Blue Hole. In the present study, the bacterial 16S rRNA gene, with a relative abundance ranging from 34.54 to 83.03%, could not be assigned at the genus level, suggesting a high abundance of unknown microorganisms in the hole, which should be studied in more detail in the further. The diversity of bacterial communities in the water from the hole was greater than that from the outer reef slope as well as from other Chinese marginal seas, such as the East China Sea, the Yellow Sea and the SCS (Guo et al., 2011; Du et al., 2013; Dong et al., 2014). In contrast, the diversity of archaeal communities in the water from the hole was lower than that from the outer reef slope as well as from other Chinese marginal seas, for instance, the Pearl Estuary, the East China Sea and the northern South China Sea (Zeng et al., 2007; Liu et al., 2014; Xie et al., 2017). A previous study showed that the water properties below 80 m in the water column were distinct from those in the upper water column in the hole (Bi et al., 2018); however, the richness and diversity of bacterial and archaeal communities showed no remarkable differences in the portions of the water column below and above 80 m in the hole (*P* > 0.05). In the present study, a sudden and dramatic increase of bacterial 16S rRNA, archaeal 16S rRNA, ANAMMOX 16S rRNA, *nirS* and *dsrB* gene copy numbers was noticed from 90 to 100 m in the hole. Meanwhile, we also found more phytoplankton at this depth compared with other depths in the hole (unpublished data). Previous studies have found a high correlation between bacteria and phytoplankton production, this is, bacterial secondary production seems to correlate well with phytoplankton biomass and primary production, probably attributed to the release of dissolved organic carbon from phytoplankton (Cole et al., 1982, 1988; Bell and Kuparinen, 1984). Thus, we hypothesized that a sudden increase of microbial abundance from 90 to 100 m in the hole may be associated with an increase of phytoplankton in this study.

*Proteobacteria*, *Cyanobacteria*, *Bacteroidetes*, *Firmicutes*, *Actinobacteria* and *SAR406* were the dominant phyla in the water from the hole and the outer reef slope, consistent with previous studies in other oceans (Malmstrom et al., 2007; Biers et al., 2009; Li et al., 2018). *SAR406*, now known as the candidate phylum *Marinimicrobia*, is ubiquitous in dark oceans, such as the northeastern subarctic Pacific Ocean (Allers et al., 2013), Arabian Sea (Fuchs et al., 2005) and Atlantic Ocean (Schattenhofer et al., 2009). *SAR406* can contribute to dark dissolved inorganic carbon fixation (Guerrero-Feijóo et al., 2018). Moreover, metagenomic and metatranscriptomic analyses have revealed a potential role of *SAR406* in carbon and dissimilatory inorganic nitrogen and sulfur cycle (Bertagnolli et al., 2017). Here, we also observed a significant difference in the abundance of *SAR406* between the hole and the outer reef slope (*P* < 0.05); therefore, we can speculate that the biogeochemical processes driven by *SAR406*, such as dark dissolved inorganic carbon fixation and dissimilatory inorganic nitrogen cycle, are probably more active in the hole than in the outer reef slope. Moreover, the abundance of *SAR406* was greater from 80 to 100 m in the water column in the hole, consistent with the finding that *SAR406* is particularly abundant in oxygen minimum zones (Wright et al., 2014).

Within *Proteobacteria*, *Deltaproteobacteria*, *Alphaproteobacteria* and *Epsilonproteobacteria* differed significantly between the hole and the outer reef slope (*P* < 0.05). *Deltaproteobacteria* is frequently found in anaerobic conditions and associated with sulfate reduction (Jørgensen and Bak, 1991; Coleman et al., 1993; MacGregor et al., 2002). Compared with the outer reef slope, the hole supported a much greater abundance of *Deltaproteobacteria*, suggesting that significant sulfate reduction occurs in the hole. *Desulfarculaceae*, *Desulfobacteraceae*, and *Desulfobulbaceae*, which are all SRB, dominated the *Deltaproteobacteria* at the family level and showed a greater abundance in the hole (*P* < 0.05), verifying our hypothesis that sulfate reduction would be more frequent in the hole. As a common bacterial group in the open ocean, *Alphaproteobacteria* was reported to be involved in dimethylsulfoniopropionate (DMSP) degradation (Moran et al., 2003). The abundance of *Alphaproteobacteria* in the water from the outer reef slope was much higher than that from the hole, indicating a more active DMSP degradation process may occur in the outer reef slope. *Rhodobacterales*, a common *Alphaproteobacteria* order in polyhaline water, was also predominant in the water from the outer reef slope. As a kind of phototrophic *Alphaproteobacteria*, *Rhodobacterales* displays a decreasing trend with depth (Imhoff et al., 2005), consistent with our results.

*Epsilonproteobacteria* is distributed in diverse natural environments, especially extreme environments such as deep-sea hydrothermal vents (Voordeckers et al., 2005; Pérez-Rodríguez et al., 2010), deep-sea volcanos (Meyer and Huber, 2014) and sulfidic aquifers (Keller et al., 2015). Most members of *Epsilonproteobacteria* are chemoautotrophs and have diverse metabolic potentials including carbon fixation, denitrification and reduced sulfur compound oxidation, playing crucial roles in deep-sea biogeochemical element cycle (Inagaki et al., 2004; Takai et al., 2006; Sievert and Vetriani, 2012; Hou et al., 2018). In our study, the abundance of *Epsilonproteobacteria* exhibited a significant difference between the portions of the water column below and above 100 m in the hole (*P* < 0.05), and its abundance sharply increased at a depth of 100 m, possibly because the hole became a sulfidic environment at a depth of 100 m (data not shown).

A previous study found that light decays at a depth of 90 m in the hole (Bi et al., 2018), probably affecting the abundance of certain microorganisms. For example, the abundance of *Cyanobacteria* and *Chlorobi* differed significantly between the portions of the water column above and below 90 m (*P* < 0.05). As oxygenic photosynthetic bacteria, *Cyanobacteria* also display endogenous respiratory metabolism in the dark at the expense of a limited range of sugars, but the growth in the dark is always slower than photoautotrophic growth, which could explain why the abundance of *Cyanobacteria* in the upper portions of the water column is much greater (Stanier and Cohen-Bazire, 1977; Whitton and Potts, 2007). In our study, the abundance of *Chlorobi* in the portions of the water column below 90 m was much greater than that in the upper waters, and its peak value was found at a depth of 100 m, which was attributed to the ability of *Chlorobi* to outcompete other phototrophs at low light intensities (Tabita and Hanson, 2004).

Discussing the inner connection among different bacterial functional groups will help us to understand the biogeochemical cycles in the hole. Previous studies have found that a strong coexistence relationship was observed between SRB and SOB in coastal marine sediments (Asami et al., 2005). In this study, we also found a significant positive correlation between SRB and SOB, indicating that sulfate reduction and sulfur oxidation process was observed to couple with each other. Moreover, both SRB and SOB were positively related to NOB, illustrating that a certain degree of coupling between sulfur cycle and nitrogen cycle in the hole. For the bacteria that participate in the nitrogen cycle in the hole, there was a significant positive correlation between denitrifier and NOB, which may suggest a synergistic effect between denitrification and nitrogen fixation process. Furthermore, SOB and denitrifier were widely distributed in every cluster and exhibited more positive correlations with other functional groups, also verify our result that sulfur oxidation and nitrate reduction were the most abundant ecological functions in the Sansha Yongle Blue Hole.

*Euryarchaeota* and *Thaumarchaeota* dominated the archaeal communities in the present study, and these two groups are the most common phyla in Archaea (Abreu et al., 2001; Stoica and Herndl, 2007; Hao et al., 2010). Some sequences related to *Nitrosopumilus* and *Nitrososphaera*, which reportedly participate in aerobic ammonia oxidation, have also been observed in the hole (Könneke et al., 2005). Thus, we speculate that aerobic ammonia oxidation is relatively high in the hole. In this study, we also observed a high proportion of *Halobacteria* members in the hole, especially in the portions of the water column below 100 m, implying that *Halobacteria* required a highly saline environment, consistent with the RDA results in which salinity explained most of the variations in the archaeal communities in the hole and *Halobacterium* was positively related to salinity (Kamekura et al., 1997; Oren, 2015).

Anchialine caves generally support complex and diverse microbial assemblages, but the microbial communities in caves were poorly understand, especially for archaea. Until now, only a few direct studies within caves have been carried out (Holmes et al., 2001; Seymour et al., 2007; Garman et al., 2011; Gonzalez et al., 2011; Humphreys et al., 2012; Pakes, 2013; Busquets et al., 2014; Davis and Garey, 2018). In this study, the other nine caves were chosen to compare the dominant groups and dominant predicted ecological functions in the Yongle Blue Hole (Table 4). *Gammaproteobacteria* and *Deltaproteobacteria* were observed to be more abundant in the deep caves, whereas *Epsilonproteobacteria* were more common in the shallow caves such as Bjejaika Cave and Lenga Pit. *Epsilonproteobacteria* occupied the absolute advantage from 100 to 150 m in the Yongle Blue Hole, which was obviously different from the other caves. As for archaea communities, the dominant group in the Bundera Sinkhole and Bjejaika Cave was *Thaumarchaeota* and *Crenarchaeota*, respectively. *Euryarchaeota* and *Thaumarthaeota* dominated in the Hospital Hole and the Yongle Blue Hole, but there were still some differences between them. In the Hospital Hole, *Thaumarthaeota* and *Euryarchaeota* dominated in the oxic and anoxic layers, respectively; whereas *Euryarchaeota* had an absolute dominance both in the oxic and anoxic layers in the Yongle Blue Hole. In brief, the bacterial dominant groups in the upper layers of the Yongle Blue Hole were similar to those in other caves, while the dominant groups in its deep layers significantly differed from others, possibly because the water columns below 100 m in the Yongle Blue Hole exhibited the thickest stable anoxic extreme conditions which was never observed in other caves in the world. Meanwhile, we also found that the archaeal community structure was quite different from other caves, probably due to its unique hydrological, geological and chemical characteristics. Studies have shown that microbes can be involved in the biogeochemical processes such as nitrogen and sulfur cycles. The dominant predicted ecological functions in different caves were similar to each other (Table 4): we observed that the bacterial dominant predicted ecological functions were sulfur oxidation and sulfate reduction, and the archaeal dominant predicted ecological functions were methanogenesis and aerobic ammonia oxidation. The highly similar dominant predicted ecological functions in different caves may be affected by their environmental conditions such as anoxic water layers and high hydrogen sulfide concentrations, indicating that environmental conditions may contribute significantly to the bacterial and archaeal communities.

**TABLE 4:** Bacterial and archaeal dominant groups and dominant predicted ecological functions in the Sansha Yongle Blue Hole and other anchialine caves.

	**Anchialine caves**	**Dominant groups**	**Dominant predicted ecological functions**	**References**
Bacteria	Bundera Sinkhole	*Deltaproteobacteria*, *Gammaproteobacteria*	Sulfate reduction, sulfur oxidation	Seymour et al., 2007; Humphreys et al., 2012
	Bjejaika Cave	*Epsilonproteobacteria*	–	Krstulović et al., 2013
	Lenga Pit	*Epsilonproteobacteria*	–	Krstulović et al., 2013
	Hospital Hole	*Halioglobus*, *Sulfurimonas*, *Escherichia*	Sulfur oxidation, sulfate reduction	Davis and Garey, 2018
	Nullarbor Cave	*Gammaproteobacteria* (*Pseudomonas*, *Pseudoalteromonas*)	Nitrite oxidation	Holmes et al., 2001
	Jewfish Sink	*Proteobacteria*	Sulfate reduction, sulfur oxidation, iron reduction	Garman et al., 2011; Rubelmann, 2014
	Bahamian blue holes	*Chlorobi*, *Deltaproteobacteria*	Sulfate reduction	Gonzalez et al., 2011
	Cenote Crustacea	*Gammaproteobacteria*, *Deltaproteobacteria*	Nitrate reduction, iron oxidation	Pakes, 2013
	Cova des Pas de Vallgornera	*Gammaproteobacteria*, *Actinobacteria*	–	Busquets et al., 2014
	Sansha Yongle Blue Hole	*Proteobacteria*	Sulfur oxidation, nitrate reduction	This study
Archaea	Bundera Sinkhole	*Thaumarchaeota*	Ammonia oxidation	Seymour et al., 2007; Humphreys et al., 2012
	Bjejaika Cave	*Crenarchaeota*	–	Krstulović et al., 2013
	Hospital Hole	*Methanococcus*, *Nitrosopumilus*	Methanogenesis, ammonia oxidation	Davis and Garey, 2018
	Jewfish Sink	*Crenarchaeota*	Methanogenesis	Garman et al., 2011; Rubelmann, 2014
	Sansha Yongle Blue Hole	*Euryarchaeota*	Methanogenesis, ammonia oxidation	This study

*–, not available.*

Different environments maintain distinctive microbial communities (Martiny et al., 2006). A dark, anaerobic, sulfidic and methane-producing condition was found below the 100 m in the water column in the hole, while the outer reef slope showed no similar environmental conditions. Previous study has found that the hole has no large-scale connection with adjacent oceans (Bi et al., 2018), thus the hole appeared to have a unique microbial community that is different from the adjacent oceans such as the outer reef slope. Sulfur oxidizers such as *Sulfurimonas* and *Thiomicrospira* genera comprised 4.5% (on average) of the bacteria present in the hole, and exhibited an increasing trend below the 100 m in the water column. While the related sequences of this functional group could hardly be found in the outer reef slope. In addition, the bacterial and archaeal diversity and dominant groups in the Yongle Blue Hole and other areas of the SCS were also compared in this study (Table 5). It was found that the bacterial diversity in the hole was greater than that in other areas of the SCS, while the archaeal diversity in the hole was lower than that in other areas of the SCS (Tseng et al., 2015; Xia et al., 2015; Li et al., 2018). Moreover, we also found that the bacterial dominant groups in the upper layers of the hole was similar to that in other areas of the SCS; whereas the bacterial dominant group in the deep layers in the hole was *Epsilonproteobacteria*, especially in the water columns from 100 to 150 m. *Epsilonproteobacteria* exhibited less abundance in other areas of the SCS, which was significantly different from the hole. As for the archaeal communities, the dominant groups in the hole and other areas of the SCS were the same at the phylum level, but differences still existed at the lower levels. For example, the dominant genus was *Halobacterium* in the hole, but Marine Group II that are involved in the methanogenesis process occupies the dominant position in other areas of the SCS. This can be due to the unique environmental conditions in the hole that are markedly different from other areas of the SCS, especially no large-scale connection with the adjacent oceans.

**TABLE 5:** Bacterial and archaeal diversity index and community structure in the Sansha Yongle Blue Hole and other areas of the South China Sea.

	**Location**	**Shannon**	**Dominant groups**	**Reference**
Bacteria	Open South China Sea	3.32 ± 0.36	upper layer: *Gammaproteobacteria*, *Alphaproteobacteria*, *Cyanobacteria* intermediate and deep layer: *Firmicutes*	Li et al., 2018
	Pelagic zones of the South China Sea	4.39 ± 0.31	Upper layer: *Alphaproteobacteria*, *Cyanobacteria* deep layer: *Gammaproteobacteria*	Tseng et al., 2015
	Northern South China Sea	4.96 ± 0.50	*Proteobacteria* (*Alphaproteobacteria*, *Gammaproteobacteria*)	Xia et al., 2015
	Sansha Yongle Blue Hole	5.10 ± 1.26	Upper layer: *Gammaproteobacteria*, *Alphaproteobacteria*, *Cyanobacteria* deep layer: *Gammaproteobacteria*, *Epsilonproteobacteria*	This study
Archaea	Pelagic zones of the South China Sea	3.55 ± 0.17	Upper layer: *Euryarchaeota* (*Marine Group II*) deep layer: *Euryarchaeota* (*Marine Group III*)	Tseng et al., 2015
	Northern South China Sea	3.02 ± 0.51	*Euryarchaeota* (*Marine Group II*), *Crenarchaeota* (*Marine Group I*)	Xia et al., 2015
	Sansha Yongle Blue Hole	1.83 ± 1.43	*Euryarchaeota* (*Halobacterium*)	This study

**FIGURE 7:**
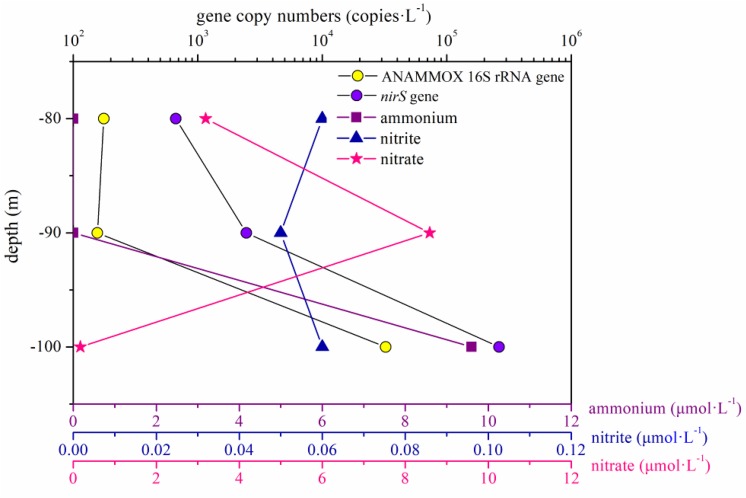
ANAMMOX 16S rRNA and *nirS* gene copy numbers and the dissolved inorganic nitrogen (ammonium, nitrite, and nitrate) concentrations in the portion of the water column between 80 and 100 m in the Sansha Yongle Blue Hole.

In the present study, the abundance of the ANAMMOX 16S rRNA and *nirS* genes showed no remarkable differences between the portions of the water column below and above 80 m in the hole (*P* > 0.05). However, the abundance of these two genes increased significantly between 80 and 100 m in the water column in the hole, especially between 90 and 100 m (Figure 7). According to previous studies, a thermocline exists within the depth range from 80 to 100 m, and the nutrient profiles vary distinctively in the thermocline (Bi et al., 2018; Yao et al., 2018). In the portions of the water column between 90 and 100 m in the hole, the nitrite and nitrate concentration slightly increased and sharply decreased, respectively, and the DO concentration reached 0 mg ⋅ L^–1^; therefore, we speculated that denitrification and ANAMMOX process would be intense in this layer. The abundance of the ANAMMOX 16S rRNA and *nirS* genes exhibited an increasing trend in this layer, confirming our hypothesis. However, the abundance of *nirS* gene was higher than that of ANAMMOX 16S rRNA gene at each depth. Moreover, considering the slow growth rates of ANAMMOX bacteria, it is hypothesized that denitrification should thus have a competitive advantage at these depths in the hole.

The environmental conditions in the hole are unique, and some environmental factors, such as hydrogen sulfide concentration and methane concentration under anaerobic conditions, may be more important to the microbial communities. Moreover, in the present study, we targeted only the bacterial and archaeal communities in the hole; the community structure of microorganisms with special ecological functions and their influence on biogeochemical processes also need to be studied further in the future.

## Conclusion

In this paper, the richness and diversity of bacterial communities in the water from the hole and the outer reef slope was greater than those of archaeal communities. Temperature and nitrate concentration significantly contributed to the heterogeneous distribution of major bacterial clades, whereas no single environmental factor significantly contributed to the archaeal communities. At such a unique environment, sulfur oxidation and nitrate reduction were predicted to be the most abundant ecological functions in the hole. The co-occurrence network analysis illustrated a synergistic effect between sulfate reduction and sulfur oxidation, and between nitrogen fixation and denitrification, a certain degree of coupling between sulfur and nitrogen cycle was observed in the hole. A sudden and dramatic increase of the five microbial groups we studied was observed from 90 to 100 m in the hole, probably due to more phytoplankton at this depth. The vertical distributions of microbial abundance were closely related to the variations in multiple environmental factors in the hole, but the relationship was non-significant. The comparisons of bacterial and archaeal communities in the hole and other caves in the world (or other areas of the South China Sea) suggest that similar conditions are hypothesized to give rise to similar microbial communities, and environmental conditions may contribute significantly to the bacterial and archaeal communities.

## Data Availability Statement

The datasets generated for this study can be found in the NCBI Sequence Read Archive (SRA) database under the accession numbers SRP152110 (https://www.ncbi.nlm.nih.gov/sra/?term=SRP152110) and SRP152191 (https://www.ncbi.nlm.nih.gov/sra/?term=SRP152191).

## Author Contributions

LiF, NB, ZY, and YZ designed the experiments. HH, LuF, and QL performed the experiments and analyzed the data. HH and YZ wrote the manuscript.

## Conflict of Interest

The authors declare that the research was conducted in the absence of any commercial or financial relationships that could be construed as a potential conflict of interest.
